# Significant Improvement of Metabolic Characteristics and Bioactivities of Clopidogrel and Analogs by Selective Deuteration

**DOI:** 10.3390/molecules21060704

**Published:** 2016-05-30

**Authors:** Xueyu Xu, Xue Zhao, Zhichao Yang, Hao Wang, Xiangjun Meng, Chong Su, Mingyuan Liu, John Paul Fawcett, Yan Yang, Jingkai Gu

**Affiliations:** 1 College of Life Sciences, Jilin University, Qianjin Street, Changchun 130012, China; x-album@163.com (X.X.); zhaoxue1310@163.com (X.Z.); yangzhichao3639@163.com (Z.Y.); wanghao340702@126.com (H.W.); 2 Research Center for Drug Metabolism, Jilin University, Qianjin Street, Changchun 130012, China; mengxj1990@163.com (X.M.); suchonghappy1@sina.com (C.S.); 3 Department of Pharmacology, School of Basic Medical Sciences, Jiamusi University, Jiamusi 154007, China; liumingyuan12@163.com; 4 School of Pharmacy, University of Otago, P.O. Box 56, Dunedin, New Zealand; paul.fawcett@otago.ac.nz; 5 Clinical Pharmacology Center, Research Institute of Translational Medicine, The First Hospital of Jilin University, Dongminzhu Street, Changchun 130061, China

**Keywords:** deuteration, clopidogrel, vicagrel, prodrug, active metabolite, antiplatelet agent

## Abstract

In the search for prodrug analogs of clopidogrel with improved metabolic characteristics and antiplatelet bioactivity, a group of clopidogrel and vicagrel analogs selectively deuterated at the benzylic methyl ester group were synthesized, characterized, and evaluated. The compounds included clopidogrel-*d*_3_ (**8**), 2-oxoclopidogrel-*d*_3_ (**9**), vicagrel-*d*_3_ (**10a**), and 12 vicagrel-*d*_3_ analogs (**10b**–**10m**) with different alkyl groups in the thiophene ester moiety. The D_3_C-O bond length in **10a** was shown by X-ray single crystal diffraction to be shorter than the H_3_C-O bond length in clopidogrel, consistent with the slower rate of hydrolysis of **8** than of clopidogrel in rat whole blood *in vitro*. A study of the ability of the compounds to inhibit ADP-induced platelet aggregation in fresh rat whole blood collected 2 h after oral dosing of rats with the compounds (7.8 μmol/kg) showed that deuteration increased the activity of clopidogrel and that increasing the size of the alkyl group in the thiophene ester moiety reduced activity. A preliminary pharmacokinetic study comparing **10a** with vicagrel administered simultaneously as single oral doses (72 μmol/kg of each drug) to male Wistar rats showed **10a** generated more of its active metabolite than vicagrel. These results suggest that **10a** is a potentially superior antiplatelet agent with improved metabolic characteristics and bioactivity, and less dose-related toxicity.

## 1. Introduction

**Scheme 1:**
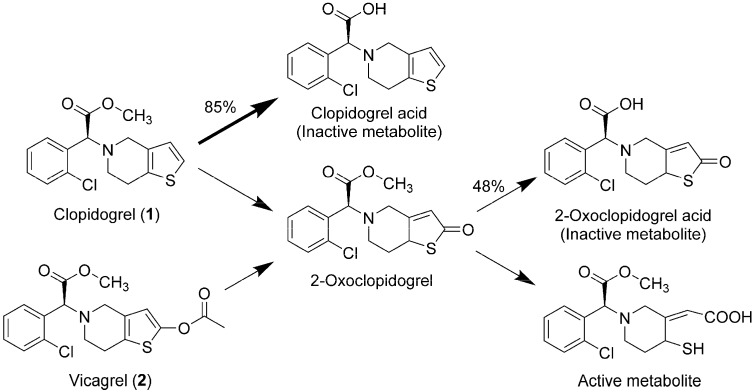
Metabolic pathway of clopidogrel and vicagrel.

Clopidogrel (**1**) is a thienopyridine antiplatelet agent approved by the US Food and Drug Administration (FDA) for the treatment of cardiovascular diseases, including atherothrombosis, unstable angina and myocardial infarction [1]. Clopidogrel is an inactive prodrug that requires conversion to an active metabolite (AM) by cytochrome P450 (P450) enzymes in the liver to exhibit an antiplatelet effect [2,3]. The AM has a thiol group that irreversibly inhibits the binding of 2MeS-ADP to P2Y12 by covalent binding to a cysteine residue in the receptor through a disulfide bond [4,5]. However, the process is inefficient for two reasons. Firstly, as shown in Scheme 1, the majority (85%) of clopidogrel is hydrolyzed by esterases to inactive clopidogrel acid [6], and secondly, only a small proportion of the remaining clopidogrel is converted to the AM by two reactions involving 2-oxoclopidogrel as an intermediate [7,8]. CYP2C19, a P450 isoform, contributes to both these reactions (44.9% to the first step, and 20.6% to the second) [9] but poor metabolizers (PMs) for CYP2C19 produce less AM with consequently little inhibition of platelets. This so-called clopidogrel resistance of PMs leads them to have a one- to five-fold higher risk of death, myocardial infarction and stroke than CYP2C19 extensive metabolizers [10,11] prompting the FDA to assign a blackbox warning to clopidogrel.

**Scheme 2:**
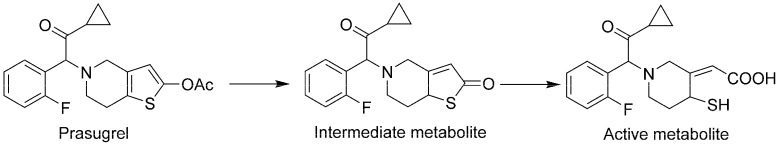
Metabolic pathway of prasugrel.

Prasugrel is a new antiplatelet agent which also exists as a prodrug that achieves greater and faster P2Y12 receptor-mediated platelet inhibition, but does not require conversion to its AM by CYP2C19. Instead it generates a thiolactone intermediate analogous to 2-oxoclopidogrel exclusively by esterase-mediated hydrolysis (Scheme 2) [12]. In addition, it has a cyclopropylketone group in place of the methyl ester in clopidogrel and, thus, neither it nor its thienolactone intermediate is deactivated via ester hydrolysis to a carboxylic acid analogous to clopidogrel acid. This means that prasugrel provides more pronounced platelet inhibition than clopidogrel with less intersubject variability. Despite these advantages, the FDA also assigned a blackbox warning to prasugrel because of its ability to cause significant, sometimes fatal, bleeding in patients with active pathological bleeding or a history of transient ischemic attack or stroke.

Inspired by prasugrel, vicagrel (**2**) was designed partially to overcome clopidogrel resistance [13]. Like prasugrel, it forms a thienolactone (in this case 2-oxoclopidogrel) by esterase-mediated hydrolysis rather than by oxidative metabolism (Scheme 1) [13,14]. However, it is also susceptible to hydrolysis by serum and intestinal esterases to analogues of clopidogrel acid and 2-oxoclopidogrel acid [15,16]. Hence, a strategy to enhance the resistance of the benzylic methyl esters of clopidogrel and vicagrel to hydrolysis has the potential to produce significantly improved antiplatelet agents.

Deuterium is a non-radioactive isotope of hydrogen that replaces it in deuterated compounds. Such deuteration causes minimal structural perturbation and has little effect on the pharmacological activity of physiologically-active compounds [17]. However, it can affect the rate of metabolism of drugs that undergo metabolism involving C–H bond scission, although the effect is highly unpredictable and dependent on the specific compound and its deuterium substitution pattern. Irrespective of whether deuteration affects the rate of metabolism, it does not appear to result in unique metabolites that are not observed for all-hydrogen analogs [18].

The subtle, but sometimes powerful, effect of deuteration has the potential to positively affect the safety, efficacy, and/or tolerability of drugs [17,19]. For example, SD-809, an analog of tetrabenazine with deuterated methoxy groups, forms an active metabolite that undergoes CYP2D6-mediated O-dealkylation at almost half the rate of the active metabolite of tetrabenazine, itself [20]. This provides a superior pharmacokinetic profile which has led to a New Drug Application (NDA) for SD-809 to the FDA for the treatment of chorea associated with Huntington’s disease.

On this basis, we envisaged that deuteration of the benzylic methyl ester group in clopidogrel and vicagrel to give clopidogrel-*d*_3_ (**8**) and vicagrel-*d*_3_ (**10a**) would reduce their susceptibility to inactivation, increase formation of their AM, and increase their antiplatelet potency. We also hypothesized that the antipode of vicagrel-*d*_3_ (***R*-10a**) would be inactive and that changing the alkyl group in the thiophene ester moiety of vicagrel-*d*_3_ would change the rate of formation of the AM with potential improvement of antiplatelet activity.

## 2. Results and Discussion

### 2.1. Chemistry

**Scheme 3:**
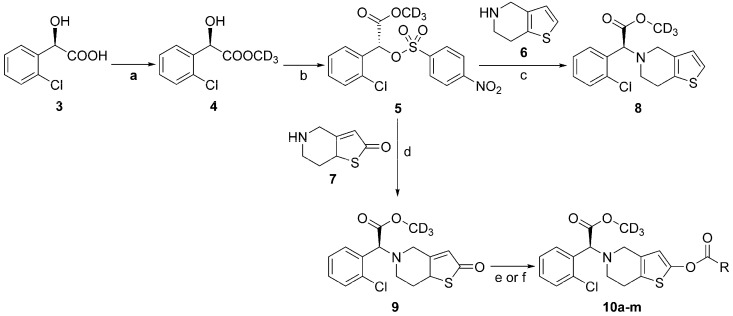
Synthesis of deuterated clopidogrel- and vicagrel-related compounds. Reagents and conditions: (**a**) Methanol-*d*_4_, HCl/dioxane, reflux; (**b**) 4-nitrobenzenesulfonyl chloride, CH_2_Cl_2_, Et_3_N, DMAP, 0 °C; (**c**) K_2_CO_3_, acetone, reflux; (**d**) K_2_CO_3_, acetonitrile, *r.t.*; (**e**) acetic anhydride, CH_2_Cl_2_, DIPEN, 0 °C to *r.t.*; and (**f**) RCOCl, CH_2_Cl_2_, DIPEN, 0 °C to *r.t.* For R, see Table 1.

Clopidogrel-*d*_3_ (**8**), 2-oxoclopidogrel-*d*_3_ (**9**), vicagrel-*d*_3_ (**10a**), and 12 vicagrel analogs (**10b**–**10m**) were synthesized by the route shown in Scheme 3. (*R*)-2-Chloromandelic acid (**3**) was dissolved in methanol-*d*_4_, and refluxed with a 1,6-dioxane solution of hydrogen chloride to afford methyl-*d*_3_ (*R*)-2-(2-chlorophenyl)-2-hydroxyacetate (**4**). Reaction of **4** with 4-nitrobenzenesulfonyl chloride in the presence of triethylamine at low temperature afforded methyl-*d*_3_ (*R*)-2-(2-chlorophenyl)-2-(4-nitrophenylsulfonyloxy) acetate (**5**). Clopidogrel-*d*_3_ (**8**) was then prepared from **5** by reaction with 4,5,6,7-tetrahydrothieno[3,2-c]pyridine hydrochloride (**6**) in acetone containing potassium carbonate. 2-Oxoclopidogrel-*d*_3_ (**9**) was prepared via reaction of **5** with 5,6,7,7a-tetrahydrothieno[3,2-*c*]pyridine-2(4*H*)-one hydrochloride (**7**) in acetonitrile containing potassium carbonate. Reaction of **9** with acetic anhydride or an acyl chloride in the presence of *N*,*N*-diisopropylethylamine (DIPEN) gave 2-acetyloxyclopidogrel-*d*_3_ (**10a**) and other acyloxyclopidogrel-*d*_3_ derivatives (**10b**−**m**) with the (*S*) configuration. The antipode of **10a** (***R*-10a**) was prepared starting from (*S*)-2-chloromandelic acid. The optical purity (% *ee*) of **8**, **10a**–**m** and ***R*-10a** was determined by chiral HPLC.

### 2.2. X-ray Single Crystal Diffraction Studies

**Figure 1:**
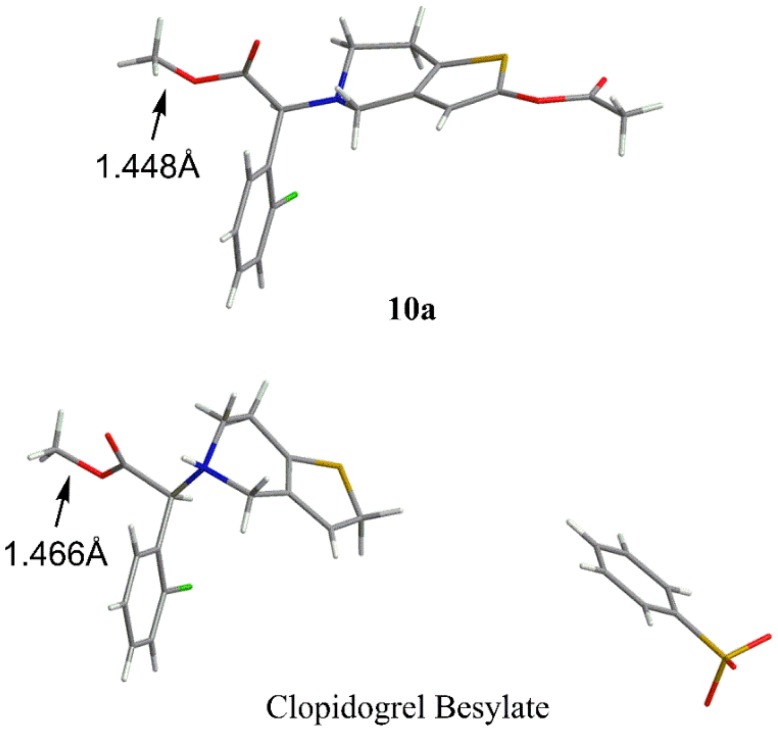
Computer-generated drawing of **10a** and clopidogrel besylate derived from X-ray single crystal diffraction data.

Single crystals of clopidogrel besylate and **10a** were subjected to X-ray Single Crystal Diffraction. Computer-generated drawings based on the results are shown on Figure 1 where it can be seen that the D_3_C-O bond length in **10a** (1.448 Å) is shorter than the corresponding H_3_C–O bond in clopidogrel besylate (1.466 Å) suggesting that the deuterated benzylic methyl ester would be more stable to esterase-mediated hydrolysis.

### 2.3. In Vitro Hydrolysis of Clopidogrel and **8** in Rat Whole Blood

**Figure 2:**
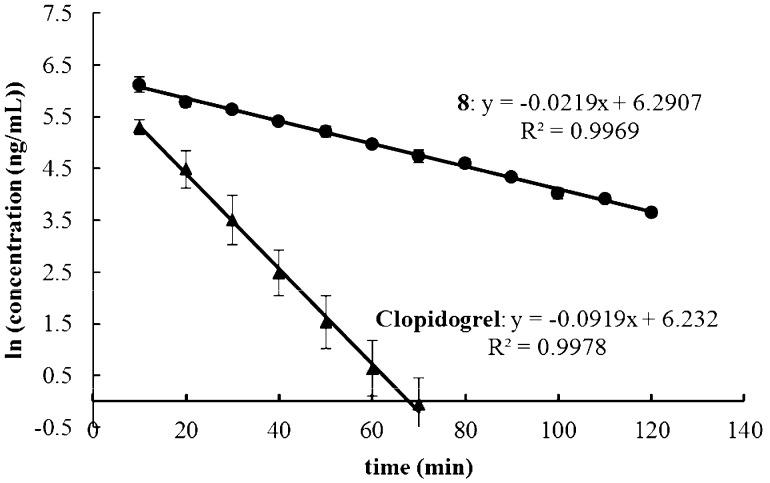
First order decomposition of clopidogrel and clopidogrel-*d*_3_ (**8**) in rat whole blood *in vitro*. Data are means ± SD, *n* = 3, for reactions at 37 °C and an initial concentration of 1000 ng/mL.

The first order rate of hydrolysis of clopidogrel-*d*_3_ (**8**) (0.0919 min^−1^) was significantly slower than that of clopidogrel (0.0219 min^−1^) in rat whole blood *in vitro* at 37 °C and an initial concentration of 1000 ng/mL (Figure 2). In fact, the concentration of clopidogrel was below the limit of detection after 70 min in contrast to that of **8** which was still detectable after 2 h. This reduced rate of hydrolysis is consistent with the results of the X-ray single crystal diffraction study reported in Section 2.2.

### 2.4. Inhibition of ADP Induced Platelet Aggregation in Rats and SAR Analysis

The antiplatelet effects of clopidogrel, vicagrel, clopidogrel-*d*_3_ (**8**), vicagrel-*d*_3_ (**10a**), 2-oxoclopidogrel-*d*_3_ (**9**), and 12-deuterated vicagrel-related compounds (**10b**–**m**) were evaluated using Born’s method to determine the inhibition of ADP-induced platelet aggregation in rat blood *ex vivo* [21]. Compounds were administered orally at a dose of 7.8 μmol/kg and compared with the effect of clopidogrel at a dose of 78 μmol/kg. The results are summarized in Table 1 where it can be seen that clopidogrel shows a strong inhibitory effect at a dose of 78 μmol/kg, but only low activity at a dose of 7.8 μmol/kg. In contrast, clopidogrel-*d*_3_ (**8**), vicagrel, and vicagrel-*d*_3_ (**10a**) are potent inhibitors at this low dose. Not surprisingly, 2-oxoclopidogrel-*d*_3_ (**9**), the metabolite of **10a**, is also a potent antiplatelet agent.

The n-alkyl R group of the thiophene ester moiety has a significant impact on antiplatelet potency with an inverse relationship between the length of the linear chain and potency in the order **10a** > **10b** > **10c** > **10g** > **10i**. In addition, compounds with linear alkyl groups appeared to be more potent than those with branched ones (e.g., **10c** > **10d** and **10g** > **10f** and **10h**) with the exception of the compound with R = cyclopropyl (**10e**) which was anomalously potent. Esters of aromatic acids (e.g., **10j**, **10k**, **10l**) appeared to be less potent than those of aliphatic acids except for the nicotinate ester **10m** which also exhibited strong potency. These results indicate that steric hindrance at the thiophene ester carboxylate group reduces the rate of formation of the AM and reduces antiplatelet potency.

These preliminary results suggest that **10a** is the most promising drug candidate of the compounds tested. Accordingly, its antipode (***R*-10a**) was synthesized in order to investigate the effect of configuration on potency. It was found that, in contrast to **10a**, ***R*-10a** was almost inactive suggesting inhibition of platelet inhibition by the AM is stereoselective. On the basis of the above results, **10a** was selected for a study of its metabolism *in vivo*.

### 2.5. Pharmacokinetic Study of AM Generated from **10a** or Vicagrel in Rat

**Figure 3:**
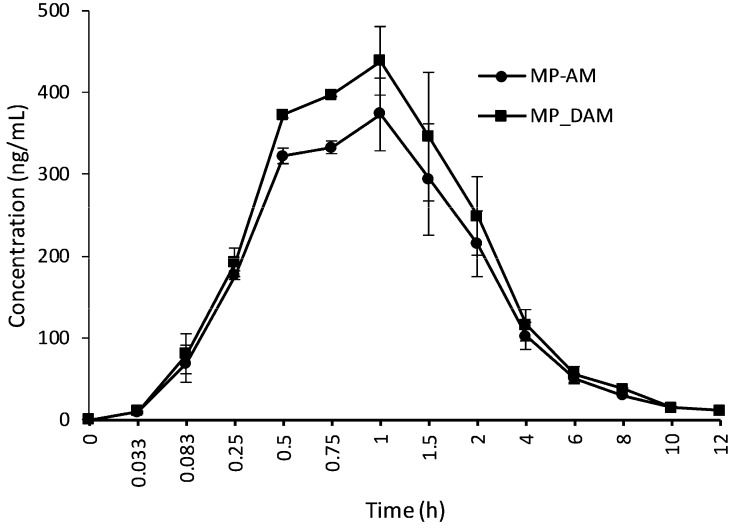
Plasma concentration-time curves of the Active Metabolites produced after simultaneous oral administration of vicagrel and **10a** to male Wistar rats at a dose of 72 μmol/kg. Each data point is expressed as mean ± SD (*n* = 3).

The AM produced from the deuterated analogs differs from that produced from clopidogrel and vicagrel only in deuteration of the benzylic methyl ester. Vicagrel has previously been shown to produce a four-fold higher level of AM than clopidogrel in rat [22] and, to compare the effect of deuteration, a pharmacokinetic study comparing the formation of the corresponding AMs from **10a** and vicagrel was carried out. In order to avoid individual differences, this involved simultaneous administration of the compounds to male Wister rats at a dose of 72 μmol/kg and collection of blood samples before the dose and at 0.033, 0.083, 0.25, 0.5, 0.75, 1, 1.5, 2, 4, 6, 8, 10, and 12 h after the dose. After reacting with 2-bromo-3′-methoxyacetophenone (MPB) to stabilize AMs [23], plasma samples were analyzed for AM derivatives from vicagrel (MP_AM) and **10a** (MP_DAM) by LC-MS/MS, which also served to confirm that the AM produced from **10a** was greater by three in molecular weight than that produced from vicagrel. At all time points, the level of MP_DAM was higher than that of MP_AM in all 3 rats (Figure 3). Corresponding pharmacokinetic parameters for vicagrel and **10a** were, respectively, C_max_ 386.0 ± 67.9 and 459.5 ± 65.5 ng/L; AUC_0–24_ 1166.5 ± 207.6 and 1346.4 ± 238.9 ng·h/L; AUC_0−∞_, 1195.7 ± 211.2 and 1389.0 ± 231.5 ng·h/L; *Vd* 47.2 ± 8.3 and 40.5 ± 6.8 L/Kg; *t*_1/2_ 2.39 ± 0.24 and 2.63 ± 0.14 h. The results indicate that the concentration of AM generated from **10a** is higher than that generated from vicagrel at the same dose.

## 3. Experimental Section

### 3.1. Materials and General Methods

Solvents and reagents were commercially available and used without further purification. Clopidogrel bisulfate and vicagrel were obtained from Hunan CHEMAPI Biological Technology Company, Changsha, Hunan, China. Melting points were uncorrected. The specific rotation of compounds was determined using a WZZ-2B automatic polarimeter (Shanghai Precision Instrument Co., Ltd., Shanghai, China). ^1^H- and ^13^C-NMR spectra were recorded on a Bruker AVANCE III 400 spectrometer (Bruker BioSpin AG, Fällanden, Switzerland). Low- and high-resolution mass spectra (LRMS and HRMS) were recorded in ESI mode using a Thermo-Scientific LTQ XL (Thermo Fisher Scientific, San Jose, CA, USA)and Waters Synapt G2 Q-TOF (Waters Corporation, Milford, MA, USA), respectively. Reactions were monitored by TLC on silica gel 60 F254 plates and column chromatography was carried out on silica gel (200–300 mesh) both from Qingdao Ocean Chemical Company, Qingdao, China. Enantiomer excess (*ee*) values were determined by chiral HPLC (Agilent Technologies, Santa Clara, CA, USA) using an Agilent 1100 system with a G1315A diode array detector. Chiral HPLC conditions are provided for each compound.

### 3.2. Synthesis of Deuterated Clopidogrel- and Vicagrel-Related Compounds

*Methyl-d_3_ (R)-2-(2-chlorophenyl)-2-hydroxyacetate* (**4**). To a stirred mixture of (*R*)-2-(2-chlorophenyl)-2-hydroxyacetic acid (**3**) (110.0 g, 0.59 mol, 99.5% *ee*) and methanol-*d*_4_ (110.0 g, 3.05 mol, 99.5% atom) at room temperature, a 1,6-dioxane solution of hydrogen chloride (3.0 mL, 3.75 mol/L) was added. The mixture was heated and refluxed for 5 h. After adding sodium bicarbonate (1.0 g), the mixture was distilled under atmospheric pressure and the methanol-*d*_4_ (74 g) recovered. The residue was mixed with dichloromethane (300 mL), washed sequentially with 5% sodium bicarbonate solution (300 mL) and brine (300 mL), dried over anhydrous sodium sulfate, and concentrated under vacuum to afford the title compound as a colorless oil (111.8 g, 93.0% yield). ^1^H-NMR (400 MHz, CDCl_3_): δ 3.69 (d, 1H, *J* = 5.2 Hz), 5.60 (d, 1H, *J* = 5.2 Hz), 7.27–7.32 (m, 2H), 7.39–7.44 (m, 2H). LRMS *m/z* 226.1 [M + Na]^+^.

*Methyl-d_3_ (R)-2-(2-Chlorophenyl)-2-(4-nitrophenylsulfonyloxy)-acetate* (**5**). To a stirred mixture of **4** (110 g, 0.540 mol), 4-nitrobenzenesulfonyl chloride (131.6 g, 0.594 mol), 2,6-dimethylpyridine (6.59 g, 0.054 mol) and dichloromethane (800 mL) at 0 °C was slowly added Et_3_N (60.6 g, 0.600 mol). After stirring for 2 h at the same temperature, the mixture was quenched with 0.5 N hydrochloric acid (400 mL). The organic layer was separated, washed with brine (800 mL), dried over anhydrous sodium sulfate, and concentrated under vacuum. The residue was recrystallized in methanol to afford the title compound as a light yellow solid (172.7 g, 82.2% yield), mp 70.8–72.1 °C, ${\left[\alpha \right]}_{D}^{20}$ −60.5 ° (c 0.5, CH_2_Cl_2_). ^1^H-NMR (400 MHz, CDCl_3_): δ 6.41 (s, 1H), 7.24–7.28 (m, 1H), 7.30*–*7.41 (m, 3H), 8.09 (d, 2H, *J* = 8.8 Hz), 8.33 (d, 2H, *J* = 8.8 Hz). LRMS *m*/*z* 411.1 [M + Na]^+^.

*Methyl-d_3_ (S)-2-(2-chlorophenyl)-2-(6,7-dihydrothieno[3,2-c]pyridin-5(4H)-yl)acetate bisulfate* (**8**). To a solution of **5** (10.00 g, 25.7 mmol) in acetone (80 mL) were added 4,5,6,7-tetrahydrothieno[3,2-*c*]pyridine hydrochloride (**6**) (4.39 g, 25.0 mmol) and potassium carbonate (7.00 g). After refluxing for 6 h, the mixture was cooled, filtered and mixed with sulfuric acid (2.60 g). After stirring for 2 h at 0 °C, the precipitate was filtered and recrystallized in purified water and acetone to afford the title compound as an off-white crystalline powder (8.88 g, 84.6% yield), mp 177.3*–*178.2 °C, ${\left[\alpha \right]}_{D}^{20}$ +44.1° (c 0.5, MeOH), 99.6% *ee* (chiral HPLC analytical conditions: Shinwa ULTRON ES-OVM, 10 μm × 4.6 mm × 250 mm, eluting with 20% acetonitrile +80% 0.01 mol/L potassium dihydrogen phosphate solution, flow rate 1.0 mL/ min, column temperature 30 °C, detection UV 220 nm). ^1^H-NMR (400 MHz, CD_3_OD): δ 3.15–3.21 (m, 2H), 3.63–3.70 (m, 1H), 3.79 (s, 1H), 4.13–4.16 (m, 1H), 4.33 (s, 1H), 5.76 (s, 1H), 6.73 (d, 1H, *J* = 5.2 Hz), 7.30 (d, 1H, *J* = 5.2 Hz), 7.42–7.46 (m, 1H), 7.49–7.60 (m, 3H). ^13^C-NMR (100 MHz, CD_3_OD): δ 167.2, 135.3, 132.7, 131.5, 130.9, 130.1, 128.5, 126.8, 126.7, 125.5, 124.8, 65.9, 48.1, 47.9, 47.7, 21.7. HRMS calculated for C_16_H_14_D_3_ClNO_2_S [M + H]^+^ *m*/*z* 325.0851, found 325.0915.

*Methyl-d_3_ (2S)-2-(2-chlorophenyl)-2-(2-oxo-2,6,7,7a-tetrahydrothieno[3,2-c]pyridin-5(4H)-yl)acetate* (**9**). To a solution of **5** (155.5 g, 0.400 mol) in acetonitrile (1000 mL), 5,6,7,7a-tetrahydrothieno[3,2-*c*]pyridin-2(4*H*)-one hydrochloride (**7**) (77.7 g, 0.405 mol) and potassium carbonate (110 g) were added. After stirring at room temperature for 22 h, the mixture was filtered and concentrated under vacuum. The residue, a dark-yellow solid, was recrystallized in ethanol to afford the title compound as a light-yellow powder (113.9 g, 83.5% yield), mp 143.9–145.0 °C. ${\left[\alpha \right]}_{D}^{20}$ +106.6 ° (c 0.5, CH_2_Cl_2_). ^1^H-NMR (400 MHz, CDCl_3_): δ 1.89 (dq, 1H, *J*_1_ = 8.8 Hz, *J*_2_ = 4.0 Hz), 2.35–2.39 (m, 1H), 2.62 (dt, 1H, *J*_1_ = 10.4 Hz, *J*_2_ = 2.0 Hz), 3.04 (d, 1H, *J* = 12.0 Hz), 3.26 (d, 1H, *J* = 12.0 Hz), 3.94 (dd, 1H, *J*_1_ = 10.8 Hz, *J*_2_ = 1.6 Hz), 4.16–4.20 (m, 1H), 4.93 (s, 1H), 6.05 (s, 1H), 7.30–7.34 (m, 2H), 7.43–7.46 (m, 1H), 7.54–7.56 (m, 1H). ^13^C-NMR (100 MHz, CDCl3): δ 198.6, 170.8, 167.1, 134.8, 132.8, 130.1, 129.8, 129.7, 127.2, 126.9, 77.2, 67.3, 51.7, 51.1, 49.6, 33.81. HRMS calculated for C_16_H_14_D_3_ClNO_3_S [M + H]^+^ *m*/*z* 341.0806, found 341.0817.

*Methyl-d_3_ (S)-2-(2-acetoxy-6,7-dihydrothieno[3,2-c]pyridin-5(4H)-yl)-2-(2chlorophenyl)acetate* (**10a**). To a stirred mixture of **9** (10.00 g, 29.3 mmol) and DIPEN (7.60 g, 58.8 mmol) in dichloromethane (100 mL), acetic anhydride (6.00 g, 58.8 mmol) was slowly added at 0 °C. The mixture was stirred for 3 h at room temperature and quenched with ice water. The organic layer was washed with 5% sodium bicarbonate solution and brine and dried over anhydrous sodium sulfate. The organic layer was concentrated under vacuum and the residue was purified by column chromatography (ethyl acetate and hexane) to afford a light yellow oil which was recrystallized from ethanol to afford the title compound as a white crystalline powder (8.35 g, 74.2% yield), mp 75.9–77.3 °C, 100% *ee*. Chiral HPLC analytical conditions: Chiralpak AD-H, 4.6 mm × 250 mm, eluting with 90% *n*-hexane and 10% isopropanol, flow rate 1.0 mL/min, column temperature 30 °C, detection UV 220 nm. ${\left[\alpha \right]}_{D}^{20}$ +24.6 ° (c 0.5, CH_2_Cl_2_). ^1^H-NMR (400 MHz, CDCl_3_): δ 2.26 (s, 3H), 2.77 (t, 2H, *J* = 5.2 Hz), 2.89 (t, 2H, *J* = 5.2 Hz), 3.60 (dd, 2H, *J*_1_ = 34.0 Hz, *J*_2_ = 14.4 Hz), 4.91 (s, 1H), 6.26 (s, 1H), 7.25–7.32 (m, 2H), 7.40–7.42 (m, 1H), 7.68–7.70 (m, 1H). ^13^C-NMR (100 MHz, CDCl_3_): δ 171.3, 167.8, 149.5, 134.7, 133.7, 129.9, 129.8, 129.4, 129.2, 127.2, 125.8, 112.0, 77.2, 67.8, 50.3, 48.1, 25.0, 20.7. HRMS calculated for C_18_H_16_D_3_NO_4_SCl [M + H]^+^ *m*/*z* 383.0912, found 383.0930.

*(S)-5-(1-(2-Chlorophenyl)-2-(methoxy-d3)-2-oxoethyl)-4,5,6,7-tetrahydrothieno[3,2-c]pyridin-2-yl propionate hydrochloride* (**10b**). To a solution of **9** (2.00 g, 5.87 mmol) and DIPEN (1.78 g, 17.6 mmol) in dichloromethane (20 mL), propionyl chloride (1.63 g, 17.6 mmol) was slowly added at 0 °C. After stirring at room temperature for 1 h, the mixture was poured into ice and 5% bicarbonate solution. The organic layer was washed with brine, dried over anhydrous sodium sulfate, and concentrated under vacuum. The residue was purified by column chromatography (ethyl acetate and hexane) to give a light yellow oil which was dissolved in ethyl ether to which 1,6-dioxane solution of hydrogen chloride (1 mL, 3.75 M) was added. The precipitate was filtered to offer the title compound as a light yellow powder (1.64 g, 64.4% yield), mp 87.9–90.3 °C, 98.7% *ee*. Chiral HPLC analytical conditions were the same as for **10a**. ^1^H-NMR (400 MHz, CDCl_3_): δ 1.27 (t, 3H, *J* = 7.6 Hz), 2.61 (q, 2H, *J* = 7.6 Hz), 2.88–3.08 (m, 2H), 3.28 (bs, 1H), 3.67–3.83 (m, 2H), 4.24 (bs, 1H), 5.55 (s, 1H), 6.39 (s, 1H), 7.43–7.52 (m, 3H), 8.31 (s, 1H), 14.1 (bs, 1H). ^13^C-NMR (100 MHz, CDCl_3_): δ 170.8, 166.3, 151.5, 135.0, 132.0, 131.0, 130.6, 130.5, 128.8, 128.7, 123.0, 110.8, 77.3, 67.1, 58.4, 53.5, 27.3, 18.4, 8.8. HRMS calculated for C_19_H_18_D_3_NO_4_SCl [M + H]^+^ *m*/*z* 397.1069, found 397.1237.

*(S)-5-(1-(2-Chlorophenyl)-2-(methoxy-d3)-2-oxoethyl)-4,5,6,7-tetrahydrothieno[3,2-c]pyridin-2-yl butyrate* (**10c**). To a solution of **9** (2.00 g, 5.87 mmol) and DIPEN (1.78 g, 17.6 mmol) in dichloromethane (20 mL), butyryl chloride (1.87, 17.6 mmol) was slowly added at 0 °C. After stirring at room temperature for 1 h, the mixture was poured into ice and 5% bicarbonate solution. The organic layer was washed with brine, dried over anhydrous sodium sulfate, and concentrated under vacuum. The residue was purified by column chromatography (ethyl acetate and hexane) to give a light yellow oil which was recrystallized from ethanol to offer the title compound as an off-white crystalline powder (0.88 g, 36.5% yield), mp 64.2–65.4 °C, 99.8% *ee*. Chiral HPLC analytical conditions were the same as for **10a**. ^1^H-NMR (400 MHz, CDCl_3_): δ 1.02 (t, 3H, *J* = 7.6 Hz), 1.77 (sext, 2H, *J* = 7.6 Hz), 2.53 (t, 2H, *J* = 7.6 Hz), 2.81 (s, 2H), 2.91 (s, 2H), 3.58 (s, 1H), 3.66 (s, 1H), 4.94 (s, 1H), 6.28 (s, 1H), 7.30–7.35 (m, 2H), 7.42–7.45 (m, 1H), 7.72 (s, 1H). ^13^C-NMR (100 MHz, CDCl_3_): δ 171.2, 170.4, 149.7, 134.7, 133.6, 130.0, 129.8, 129.5, 127.2, 125.6, 111.7, 100.0, 77.2, 67.8, 50.3, 48.2, 35.8, 25.0, 18.2, 13.6. HRMS calculated for C_20_H_20_D_3_NO_4_SCl [M + H]^+^ *m*/*z* 411.1225, found 411.1292.

*(S)-5-(1-(2-Chlorophenyl)-2-(methoxy-d3)-2-oxoethyl)-4,5,6,7-tetrahydrothieno[3,2-c]pyridin-2-yl isobutyrate hydrochloride* (**10d**). A similar procedure to that described for the preparation of **10b** was followed, except that an equivalent amount of isobutyryl chloride was used in place of propionyl chloride. The title compound was obtained as a white powder (1.94 g, 73.9% yield), mp 146.7–149.9 °C, 99.2% *ee*. Chiral HPLC analytical conditions were the same as for **10a**. ^1^H-NMR (400 MHz, CDCl_3_): δ 1.30 (d, 6H, *J* = 6.8 Hz), 2.80 (hept, 2H, *J* = 6.8 Hz), 3.04–4.76 (m, 6H), 5.56 (s, 1H), 6.39 (s, 1H), 7.42–7.51 (m, 3H), 8.32 (d, 1H, *J* = 6.0 Hz), 14.08 (bs, 1H). ^13^C-NMR (100 MHz, CDCl_3_): δ 173.3, 166.2, 151.8, 134.9, 132.1, 131.1, 130.5, 128.8, 126.7, 122.8, 121.7, 110.6, 77.3, 62.6, 53.5, 53.4, 33.9, 19.9, 18.7. HRMS calculated for C_20_H_20_D_3_NO_4_SCl [M + H]^+^ *m*/*z* 411.1225, found 411.1299.

*(S)-5-(1-(2-Chlorophenyl)-2-(methoxy-d3)-2-oxoethyl)-4,5,6,7-tetrahydrothieno[3,2-c]pyridin-2-yl cyclopro-panecarboxylate* (**10e**). A similar procedure to that described for the preparation of **10c** as followed, except that an equivalent amount of cyclopropanecarbonyl chloride was used in place of butyryl chloride. The title compound was obtained as a white powder (0.70 g, 29.2% yield), mp 58.4–60.8 °C, 99.7% *ee*. Chiral HPLC analytical conditions were the same as for **10a**. ^1^H-NMR (400 MHz, CDCl_3_): δ 1.02–1.07 (m, 2H), 1.16–1.20 (m, 2H), 1.78–1.84 (m, 1H), 2.79 (t, 2H, *J* = 5.6 Hz), 2.89 (t, 2H, *J* = 5.2 Hz), 3.61 (dd, 2H, *J*_1_ = 36.8 Hz, *J*_2_ = 14.4 Hz), 4.92 (s, 1H), 6.28 (s, 1H), 7.26–7.34 (m, 2H), 7.42–7.44 (m, 1H), 7.70 (dd, 1H, *J*_1_ = 5.6 Hz, *J*_2_ = 1.6 Hz). ^13^C-NMR (100 MHz, CDCl_3_): δ 171.9, 171.2, 149.8, 134.7, 133.6, 129.9, 129.8, 129.5, 129.0, 127.2, 125.6, 111.7, 77.3, 67.8, 50.4, 48.2, 24.9, 12.7, 9.7. HRMS calculated for C_20_H_18_D_3_NO_4_SCl [M + H]^+^ *m*/*z* 409.1069, found 409.1157.

*(S)-5-(1-(2-Chlorophenyl)-2-(methoxy-d3)-2-oxoethyl)-4,5,6,7-tetrahydrothieno[3,2-c]pyridin-2-yl pivalate* (**10f**). A similar procedure to that described for the preparation of **10c** was followed, except that an equivalent amount of pivaloyl chloride was used in place of butyryl chloride. The title compound was obtained as a light yellow powder (1.57 g, 62.9% yield), mp 105.4–107.1 °C, 99.4% *ee*. Chiral HPLC analytical conditions were the same as for **10a**. ^1^H-NMR (400 MHz, CDCl_3_): δ 1.33 (s, 9H), 2.80 (t, 2H, *J* = 5.2 Hz), 2.90 (t, 2H, *J* = 4.4 Hz), 3.61 (dd, 2H, *J*_1_ = 36.4 Hz, *J*_2_ = 14.0 Hz), 4.93 (s, 1H), 6.28 (s, 1H), 7.27–7.34 (m, 2H), 7.42–7.44 (m, 1H), 7.71 (d, 1H, *J* = 6.0 Hz). ^13^C-NMR (100MHz, CDCl_3_): δ 175.2, 171.2, 150.1, 134.7, 133.7, 129.9, 129.8, 129.5, 129.0, 127.2, 125.6, 111.4, 77.3, 67.8, 50.4, 48.2, 39.1, 27.0, 24.9. HRMS calculated for C_21_H_22_D_3_NO_4_SCl [M + H]^+^ *m*/*z* 425.1382, found 411.1472.

*(S)-5-(1-(2-Chlorophenyl)-2-(methoxy-d3)-2-oxoethyl)-4,5,6,7-tetrahydrothieno[3,2-c]pyridin-2-yl hexanoate* (**10g**). A similar procedure to that described for the preparation of **10c** was followed, except that an equivalent amount of hexanoyl chloride was used in place of butyryl chloride. The title compound was obtained as a white powder (0.89 g, 34.5% yield), mp 49.4–52.7 °C, 99.8% *ee*. Chiral HPLC analytical conditions were the same as for **10a**. ^1^H-NMR (400 MHz, CDCl3): δ 0.93 (t, 3H, *J* = 7.2 Hz), 1.32–1.41 (m, 4H), 1.73 (quint, 2H, *J* = 7.2 Hz), 2.53 (t, 2H, *J* = 7.2 Hz), 2.79 (t, 2H, *J* = 4.8 Hz), 2.90 (t, 2H, *J* = 5.2 Hz), 3.61 (dd, 2H, *J*_1_ = 36.0 Hz, *J*_2_ = 14.4 Hz), 4.92 (s, 1H), 6.28 (s, 1H), 7.26–7.34 (m, 2H), 7.42–7.44 (m, 1H), 7.70 (dd, 1H, *J*_1_ = 4.8 Hz, *J*_2_ = 2.0 Hz). ^13^C-NMR (100 MHz, CDCl_3_): δ 171.3, 170.7, 149.7, 134.7, 133.7, 129.9, 129.8, 129.5, 129.2, 127.2, 125.7, 111.7, 77.3, 67.9, 50.4, 48.2, 33.9, 31.2, 25.0, 24.4, 22.3, 13.9. HRMS calculated for C_22_H_24_D_3_NO_4_SCl [M + H]^+^ *m*/*z* 439.1538, found 439.1651.

*(S)-5-(1-(2-Chlorophenyl)-2-(methoxy-d3)-2-oxoethyl)-4,5,6,7-tetrahydrothieno[3,2-c]pyridin-2-yl 2,2-dimethylbutanoate* (**10h**). A similar procedure to that described for the preparation of **10c** was followed, except that an equivalent amount of 2,2-dimethylbutanoyl chloride was used in place of butyryl chloride. The title compound was obtained as a white powder (1.85 g, 71.8% yield), mp 99.4–100.3 °C, 99.8% *ee*. Chiral HPLC analytical conditions were the same as those for **10a**. ^1^H-NMR (400 MHz, CDCl_3_): δ 0.91 (t, 3H, *J* = 7.6 Hz), 1.28 (s, 6H), 1.79 (q, 2H, *J* = 7.6 Hz), 2.79 (t, 2H, *J* = 5.2 Hz), 2.90 (t, 2H, *J* = 4.8 Hz), 3.61 (dd, 2H, *J*_1_ = 34.8 Hz, *J*_2_ = 14.4 Hz), 4.92 (s, 1H), 6.28 (s, 1H), 7.26–7.33 (m, 2H), 7.41–7.44 (m, 1H), 7.70 (dd, 1H, *J*_1_ = 5.2 Hz, *J*_2_ = 2.0 Hz). ^13^C-NMR (100 MHz, CDCl_3_): δ 174.8, 171.3, 150.0, 134.7, 133.8, 129.9, 129.8, 129.5, 129.1, 127.2, 125.6, 111.5, 77.3, 67.8, 50.4, 48.2, 43.1, 33.4, 25.0, 24.5, 9.3. HRMS calculated for C_22_H_24_D_3_NO_4_SCl [M + H]^+^ *m*/*z* 439.1538, found 439.1628.

*(S)-5-(1-(2-Chlorophenyl)-2-(methoxy-d3)-2-oxoethyl)-4,5,6,7-tetrahydrothieno[3,2-c]pyridin-2-yl laurate* (**10i**). A similar procedure to that described for the preparation of **10c** was followed, except that an equivalent amount of lauroyl chloride was used in place of butyryl chloride. The title compound was obtained as a white crystalline powder (0.72 g, 23.4% yield), mp 35.8–37.6 °C, 99.7% *ee*. Chiral HPLC analytical conditions were the same as those for **10a**. ^1^H-NMR (400 MHz, CDCl_3_): δ 0.90 (t, 3H, *J* = 7.2 Hz), 1.25–1.39 (m, 16H), 1.73 (quint, 2H, *J* = 7.6 Hz), 2.53 (t, 2H, *J* = 7.6 Hz), 2.79 (s, 2H), 2.90 (s, 2H), 3.61 (dd, 2H, *J*_1_ = 35.6 Hz, *J*_2_ = 14.4 Hz), 4.92 (s, 1H), 6.28 (s, 1H), 7.28–7.34 (m, 2H), 7.42–7.44 (m, 1H), 7.71 (d, 1H, *J* = 6.0 Hz). ^13^C-NMR (100 MHz, CDCl_3_): δ 170.7, 155.1, 149.7, 134.7, 133.7, 129.9, 129.8, 129.5, 127.2, 125.7, 125.6, 111.7, 77.2, 67.9, 50.4, 48.2, 34.0, 31.9, 29.7, 29.6, 29.4, 29.3, 29.2, 29.0, 25.0, 24.7, 22.7, 14.1. HRMS calculated for C_28_H_36_D_3_NO_4_SCl [M + H]^+^ *m*/*z* 523.2477, found 523.2624.

*(S)-5-(1-(2-Chlorophenyl)-2-(methoxy-d3)-2-oxoethyl)-4,5,6,7-tetrahydrothieno[3,2-c]pyridin-2-yl cinnamate* (**10j**). A similar procedure to that described for the preparation of **10c** was followed, except that an equivalent amount of cinnamoyl chloride was used in place of butyryl chloride. The title compound was obtained as a yellow powder (1.12 g, 40.5% yield), mp 121.7–123.1 °C, 99.8% *ee*. Chiral HPLC analytical conditions were the same as those for **10a**. ^1^H-NMR (400 MHz, CDCl_3_): δ 2.85 (s, 2H), 2.98 (s, 2H), 3.66 (s, 1H), 3.73–3.76 (m, 1H), 5.00 (s, 1H), 6.39 (s, 1H), 6.59 (d, 1H, *J* = 16.0 Hz), 7.31–7.36 (m, 2H), 7.44 (s, 1H), 7.46 (s, 3H), 7.59 (t, 2H, *J* = 4.0 Hz), 7.79 (s, 1H), 7.88 (d, 1H, *J* = 16.0 Hz). ^13^C-NMR (100 MHz, CDCl_3_): δ 171.3, 163.7, 149.7, 147.4, 134.7, 134.0, 133.7, 131.0, 130.0, 129.8, 129.5, 129.1, 129.0, 128.4, 127.2, 125.9, 116.0, 111.8, 77.3, 67.8, 50.4, 48.2, 25.0. HRMS calculated for C_25_H_20_D_3_NO_4_SCl [M + H]^+^ *m*/*z* 471.1225, found 471.1328.

*(S)-5-(1-(2-Chlorophenyl)-2-(methoxy-d3)-2-oxoethyl)-4,5,6,7-tetrahydrothieno[3,2-c]pyridin-2-yl benzoate hydrochloride* (**10k**). A similar procedure similar to that described for the preparation of **10b** was followed, except that an equivalent amount of benzoyl chloride was used in place of propionyl chloride. The title compound was obtained as an off-white powder (1.04 g, 39.8% yield), mp 158.7–161.9 °C, 97.6% *ee*. Chiral HPLC analytical conditions were the same as for **10a**. ^1^H-NMR (400 MHz, CDCl_3_): δ 3.12–4.85 (m, 6H), 5.63 (s, 1H), 6.57 (s, 1H), 7.44–7.56 (m, 5H), 7.68 (t, 1H, *J* = 7.6 Hz), 8.17 (d, 2H, *J* = 7.6 Hz), 8.37 (d, 1H, *J* = 7.6 Hz), 14.18 (bs, 1H). ^13^C-NMR (100 MHz, CDCl_3_): δ 166.1, 163.0, 151.8, 135.0, 134.4, 132.3, 131.3, 131.2, 130.6, 130.3, 128.9, 128.8, 127.8, 126.5, 123.1, 111.1, 77.3, 68.3, 59.4, 53.5, 19.8. HRMS calculated for C_23_H_18_D_3_NO_4_SCl [M + H]^+^ *m*/*z* 445.1069, found 445.1172.

*(S)-5-(1-(2-Chlorophenyl)-2-(methoxy-d3)-2-oxoethyl)-4,5,6,7-tetrahydrothieno[3,2-c]pyridin-2-yl 2-(trifluoromethyl)benzoate* (**10l**). A similar procedure to that described for the preparation of **10c** was followed, except that an equivalent amount of 2-(trifluoromethyl)benzoyl chloride was used in place of butyryl chloride. The title compound was obtained as an off-white powder (1.37 g, 45.5% yield), mp 74.3–76.2 °C, 98.6% *ee*. Chiral HPLC analytical conditions were the same as for **10a**. ^1^H-NMR (400 MHz, CDCl_3_): δ 2.84 (s, 2H), 2.94 (s, 2H), 3.66 (dd, 2H, *J*_1_ = 30.8 Hz, *J*_2_ = 13.6 Hz), 4.96 (s, 1H), 6.47 (s, 1H), 7.30–7.35 (m, 2H), 7.43–7.45 (m, 1H), 7.68–7.73 (m, 3H), 7.82–7.86 (m, 1H), 7.96–7.99 (m, 1H). ^13^C-NMR (100 MHz, CDCl_3_): δ 170.8, 163.2, 149.4, 134.7, 132.2, 131.9, 131.0, 130.0, 129.9, 129.7, 129.5, 129.2, 129.0, 127.3, 127.1, 127.0, 124.5, 121.8, 112.4, 77.2, 67.5, 50.2, 48.2, 24.7. HRMS calculated for C_24_H_17_D_3_ClF_3_NO_4_S [M + H]^+^ *m*/*z* 513.942, found 513.1071.

*(S)-5-(1-(2-Chlorophenyl)-2-(methoxy-d3)-2-oxoethyl)-4,5,6,7-tetrahydrothieno[3,2-c]pyridin-2-yl nicotinate* (**10m**). A similar procedure to that described for the preparation of **10c** was followed, except that an equivalent amount of nicotinoyl chloride was used in place of butyryl chloride. The title compound was obtained as an off-white powder (0.91 g, 34.7% yield), mp 92.1–94.8 °C, 99.4% *ee*. Chiral HPLC analytical conditions were the same as for **10a**. ^1^H-NMR (400 MHz, CDCl_3_): δ 2.84 (t, 2H, *J* = 5.2 Hz), 2.95 (t, 2H, *J* = 5.2 Hz), 3.67 (dd, 2H, *J*_1_ = 33.2 Hz, *J*_2_ = 14.4 Hz), 4.96 (s, 1H), 6.48 (s, 1H), 7.30–7.35 (m, 2H), 7.43–7.50 (m, 2H), 7.72 (dd, 1H, *J*_1_ = 5.2 Hz, *J*_2_ = 1.6 Hz), 8.42 (dt, 1H, *J*_1_ = 8.0 Hz, *J*_2_ = 1.6 Hz), 8.85–8.88 (m, 1H), 9.36 (s, 1H). ^13^C-NMR (100 MHz, CDCl_3_): δ 171.2, 162.3, 154.3, 151.4, 149.3, 137.6, 134.8, 133.5, 130.0, 129.9, 129.6, 129.2, 127.3, 126.4, 124.7, 123.5, 112.5, 77.3, 67.7, 50.3, 48.1, 25.0. HRMS calculated for C_22_H_16_D_3_ClN_2_NaO_4_S [M + Na]^+^ *m*/*z* 468.0841, found 468.0950.

*Methyl-d3 (R)-2-(2-acetoxy-6,7-dihydrothieno[3,2-c]pyridin-5(4H)-yl)-2-(2chlorophenyl)acetate* (***R*-10a**). A similar procedure to that described for the preparation of **10a** was followed, except that an equivalent amount of methyl-*d*_3_ (2*R*)-2-(2-chlorophenyl)-2-(2-oxo-2,6,7,7a-tetrahydrothieno[3,2-c]pyridin-5(4*H*)-yl) acetate was used in place of **9**. The title compound was obtained as an off-white crystalline powder (5.78 g, 52.1% yield), mp 73.8–75.7 °C, 100% *ee*. Chiral HPLC analytical conditions were the same as for **10a**. ${\left[\alpha \right]}_{D}^{20}$ −24.6 ° (c 0.5, CH_2_Cl_2_). ^1^H-NMR (400 MHz, CDCl_3_): δ 2.29 (s, 3H), 2.80 (s, 2H), 2.91 (s, 2H), 3.62 (dd, 2H, *J*_1_ = 32.4 Hz, *J*_2_ = 12.0 Hz), 4.94 (s, 1H), 6.28 (s, 1H), 7.29–7.34 (m, 2H), 7.42–7.44 (m, 1H), 7.72 (s, 1H). ^13^C-NMR (100 MHz, CDCl_3_): δ 171.2, 167.8, 149.6, 134.7, 133.6, 129.9, 129.8, 129.5, 129.2, 127.2, 125.8, 112.0, 77.3, 67.8, 50.3, 48.2, 25.0, 20.7. HRMS calculated for C_18_H_16_D_3_NO_4_SCl [M + H]^+^ *m*/*z* 383.0912, found 383.0927.

### 3.3. Cultivation and X-ray Diffraction of Single Crystals of **10a** and Clopidogrel Besylate

After dissolving in ethanol, single crystals of **10a** were obtained by the slow solvent evaporation method. Clopidogrel bisulfate was suspended in dichloromethane and the mixture slowly mixed with 5% sodium bicarbonate. The organic layer was separated, dried over anhydrous sodium sulfate and concentrated under vacuum. The residue was dissolved in ethyl ether, after which an ethyl ether solution of besylic acid was added to afford a precipitate of clopidogrel besylate. After dissolving the clopidogrel besylate in isopropanol, the solution in a glass tube was placed in a sealed container with tert-butyl methyl ether to afford single crystals of clopidogrel besylate by the solvent diffusion method. X-ray single crystal diffraction of **10a** was carried out on a Rigaku MM-007 Saturn 70 instrument.

### 3.4. In Vitro Hydrolysis of Clopidogrel and **8** in Rat Whole Blood

After equilibrating at 37 °C, a normal saline solution of clopidogrel or **8** (100 μL, 30 μg/mL) was added to each of three glass test tubes of fresh whole rat blood (3 mL). Blood samples (100 μL) were collected every 10 min over 120 min, mixed with methanol (950 μL), water (50 μL) and an internal standard (IS) solution (diazepam, 100 μL, 100 ng/mL) and centrifuged at low temperature at 13,000 rpm. The supernatants (20 μL) were collected and analyzed for clopidogrel or **8** by LC-MS/MS. This was carried out using an Agilent 1100 Series HPLC system (degasser, pump, auto sampler, and column oven, Agilent Technologies, Santa Clara, CA, USA) coupled to an AB SCIEX Qtrap 2000 mass spectrometer (Applied Biosystems Sciex, Ottawa, ON, Canada) with an ESI source operated in the positive ion mode. Data acquisition and integration were performed by Analyst software (version 1.3.2, Applied Biosystems Sciex, Ottawa, ON, Canada). Chromatography was performed on a ZORBAX Extend-C18 column (150 mm × 4.6 mm i.d., 5 μm, Agilent Technologies, Santa Clara, CA, USA) maintained at 40 °C using methanol and 0.1% aqueous formic acid (20:80, *v*/*v*) as mobile phase delivered at a flow rate of 1.0 mL/min. The injection volume was 20 μL. A 50:50 split of the column eluent was made by means of a T-piece. The mass spectrometer was operated at unit resolution for Q1 and Q3 in the multiple reaction monitoring (MRM) mode with a dwell time of 200 ms per MRM channel. Clopidogrel, **8** and IS were monitored using the transitions of the protonated doubly charged molecular ions at *m/z* 324.9→214.8, *m*/*z* 321.8→211.9 and *m/z* 285.2→193.1, respectively. Optimized source-dependent parameters were as follows: ion spray voltage 4000 V; turbo heater temperature 400 °C; curtain, nebulizer and turbo gases N_2_ at 20, 50, and 45 psi, respectively; decluttering potentials and collision energies were, respectively, 40 V and 19 eV for clopidogrel, 40 V and 20 eV for **8**, and 80 V and 42 eV for IS. Stock solutions of clopidogrel or **8** (1.0 mg/mL) were prepared in methanol. Calibration standards were prepared by spiking blank rat blood at concentrations of 1, 3, 10, 30, 100, 300, and 1000 ng/mL for clopidogrel or 8. QC samples were prepared independently at concentrations of 3, 30, and 800 ng/mL in the same way. A stock solution of IS (1.0 mg/mL) was prepared in methanol and diluted with methanol and water (50:50, *v*/*v*) to give a 100 ng/mL IS working solution. Stock solutions were kept at 4 °C and calibration standards and QC samples at −80 °C when not in use.

### 3.5. Inhibition of ADP Induced Platelet Aggregation in Rats ex Vivo

After acclimatizing to a 12 h/12 h light/dark cycle and receiving regular at chow and water, male Wistar rats (weight 230–250 g) (Vital River Laboratories, Beijing, China) were divided into 20 groups of six rats each. CMC 0.25% suspensions of compounds were orally administered to rats at a dose of 7.8 μmol/kg. Clopidogrel bisulfate was used as an active control at a dose of 78 μmol/kg. CMC 0.25% was used as vehicle. Two hours after administration, rats were anesthetized using sodium pentobarbital and blood samples collected from the abdominal aorta for platelet aggregation tests using Born’s method [21]. ADP (1.0 μM, Sigma-Aldrich, Co., St. Louis, MO, USA) was used as agonist for platelet aggregation. Platelet aggregation was measured using a coagulation analyzer (NJ4, Beijing Precil Instrument Company, Beijing, China).

### 3.6. Pharmacokinetic Study of AM Generated from **10a** or Vicagrel in Rat

After a 12 h fast with free access to drinking water, three male Wistar rats (weight 180−220 g) (Vital River Laboratories, Beijing, China) were orally administrated a suspension of **10a** and vicagrel together in 0.25% CMC at equimolar doses of 27.6 mg/kg and 27.3 mg/kg respectively. Blood samples were collected into heparinized anticoagulant tubes from the fundus venous plexus before dosing, and at 0.033, 0.083, 0.25, 0.5, 0.75, 1, 1.5, 2, 4, 6, 8, 10, and 12 h after dosing. To blood sample (50 μL), acetonitrile-water (50 μL, 1:1), ammonium bicarbonate buffer (100 μL, 200 mM), 2-bromo-3′-methoxyacetophenone (MPB) (100 μL, 100 mM) and biphenyldicarboxylate as IS (50 μL, 25 ng/mL) were added. After being shaken for 10 min, a 1% formic acid in acetonitrile solution (650 μL) was added and the mixture centrifuged at low temperature at 13,000 rpm for 5 min. The supernatants (20 μL) were collected and analyzed by LC-MS/MS using the same system as Section 3.4. Chromatography was performed on an Ascentis C18 column (5 cm × 4.6 mm i.d., 5 μm, Sigma-Aldrich Co. LLC, Bellefonte, PA, USA) maintained at 40 °C using 0.1% aqueous formic acid and acetonitrile (55:45, *v*/*v*) as mobile phase delivered at a flow rate of 1.0 mL/min. The injection volume was 20 μL. A 50:50 split of the column eluent was made by means of a T-piece. The mass spectrometer was operated at unit resolution for Q1 and Q3 in the MRM mode with a dwell time of 200 ms per MRM channel. MP_AM MP_DAM and the IS were monitored using the transitions of the protonated doubly charged molecular ions at *m/z* 504.1→354.1, *m/z* 507.1→357.1 and *m/z* 436→386.9, respectively. Optimized source-dependent parameters were as follows: ion spray voltage 5500 V; turbo heater temperature 500 °C; curtain, nebulizer and turbo gases N2 at 25, 45, and 40 psi, respectively; decluttering potentials and collision energies were respectively 10 V and 10 eV. Stock solutions of MP_AM (1.0 mg/mL) were prepared in acetonitrile. Calibration standards were prepared by spiking blank rat blood at concentrations of 0.2, 0.5, 1, 3, 10, 30, 100, and 300 ng/mL for MP_AM. QC samples were prepared independently at concentrations of 0.5, 24, and 240 ng/mL in the same way. A stock solution of IS (1.0 mg/mL) was prepared in methanol and diluted with acetonitrile and water (50:50, *v*/*v*) to give a 25 ng/mL IS working solution. Stock solutions were kept at 4 °C and calibration standards and QC samples at −80 °C when not in use.

## 4. Conclusions

Although new antiplatelet agents, such as prasugrel, are now in clinical use, clopidogrel remains the most widely used antiplatelet agent because of its greater safety and lower risk of bleeding. However, it still has drawbacks, including reduced effectiveness in CYP2C19 PMs (clopidogrel resistance) and the fact that more than 90% of an oral dose is metabolized to inactive metabolites [15,16] with the potential for dose-related toxicity. The similar prodrug vicagrel [13] partially overcomes the reduced effectiveness of clopidogrel in CYP2C19 PMs, but remains susceptible to inactivation due to esterase-mediated hydrolysis [14]. In the present study, a number of selectively-deuterated clopidogrel and vicagrel-related compounds were synthesized and evaluated with the aim of increasing the resistance of the hydrolytically-susceptible benzylic methyl ester to enzyme-mediated hydrolysis and, thereby, enhancing the formation of the AM. Selective deuteration is known to produce minimal structural modification and retain pharmacological activity, but potentially modify metabolic fate [19]. Encouragingly, X-ray single crystal diffraction data showed the D_3_C-O bond length in **10a** was slightly shorter than in clopidogrel and the rate of hydrolysis of clopidogrel-*d*_3_ in rat whole blood *in vitro* was significantly slower than that of clopidogrel.

The compounds were evaluated for their inhibitory effect on ADP-induced platelet aggregation in rat whole blood *ex vivo* and some of them (e.g., **10a**, **10b**, **10c**, **10d**, **10e**, **10g**, and **10m**) shown to exhibit potent antiplatelet activity. The most active **10a** was selected to undergo a pharmacokinetic study *in vivo* to compare its ability to form its AM with that of vicagrel. After simultaneous oral administration, the concentration of AM generated from **10a** was higher than that generated from vicagrel at the same dose suggesting **10a** would produce significantly more of its AM than clopidogrel *in vivo*.

On the basis of these results, selective deuteration of the benzylic methyl ester group in vicagrel produces a potentially superior antiplatelet agent to clopidogrelwith better metabolic characteristics and bioactivity and less dose-related toxicity. Further preclinical studies of **10a** are currently underway in our laboratory to consolidate this prediction.

## Figures and Tables

**Table 1:** Inhibitory effects of clopidogrel- and vicagrel-related compounds on ADP-induced platelet aggregation in rat *ex vivo* ^a^.

Compound	R in 10a–m	Platelet Aggregation (%)
clopidogrel		15.8 ± 8.0 ^b^
		59.8 ± 8.4
vicagrel		20.0 ± 9.1
**8**		36.6 ± 6.6
**10a**	methyl	17.3 ± 8.2
**10b**	ethyl	24.9 ± 9.7
**10c**	propyl	29.9 ± 9.5
**10d**	isopropyl	36.3 ± 4.5
**10e**	cyclopropyl	24.6 ± 10.0
**10f**	tert-butyl	44.7 ± 4.1
**10g**	pentyl	35.2 ± 7.7
**10h**	tert-pentyl	48.5 ± 6.7
**10i**	undecyl	49.7 ± 4.0
**10j**	styryl	56.4 ± 6.9
**10k**	phenyl	43.6 ± 9.5
**10l**	2-(trifluoromethyl)phenyl	49.2 ± 4.6
**10m**	pyridin-3-yl	37.4 ± 6.7
***R*-10a**		57.4 ± 5.0
**9**		21.2 ± 7.6
vehicle		64.6 ± 4.9

^a^ Aggregation data are means ± SD (*n* = 6) measured *ex vivo* 2 h after oral administration of compound at a dose of 7.8 μmol/kg. *p* < 0.01 *vs.* vehicle; ^b^ Clopidogrel bisulfate used as active control at a dose of 78 μmol/kg.

## Abbreviations

The following abbreviations are used in this manuscript:

AM	active metabolite
P450	cytochrome P450
ADP	adenosine diphosphate
PMs	poor metabolizers
*ee*	enantiomer excess
DIPEN	*N*,*N*-diisopropylethylamine
IS	internal standard
MPB	2-bromo-3′-methoxyacetophenone
MP_AM	the MPB derivative of the AM
MP_DAM	the MPB derivative of the AM of **10a**
